# Dual n-back working memory training evinces superior transfer effects compared to the method of loci

**DOI:** 10.1038/s41598-021-82663-w

**Published:** 2021-02-04

**Authors:** Wenjuan Li, Qiuzhu Zhang, Hongying Qiao, Donggang Jin, Ronald K. Ngetich, Junjun Zhang, Zhenlan Jin, Ling Li

**Affiliations:** Key Laboratory for NeuroInformation of Ministry of Education, High-Field Magnetic Resonance Brain Imaging Key Laboratory of Sichuan Province, Center for Psychiatry and Psychology, School of Life Science and Technology, University of Electronic Science and Technology of China, Chengdu, 610054 China

**Keywords:** Human behaviour, Working memory

## Abstract

Working memory (WM) training is a prevalent intervention for multiple cognitive deficits, however, the transfer effects to other cognitive tasks from gains in WM induced by different training techniques still remains controversial. Therefore, the current study recruited three groups of young adults to investigate the memory training transference, with N-back group (NBG) (n = 50) training on dual n-back task, Memory Palace group (MPG) (n = 50) on method of loci, and a blank control group (BCG) (n = 48) receiving no training. Our results showed that both training groups separately improved WM capacity on respective trained task. For untrained tasks, both training groups enhanced performance on digit-span task, while on change detection task, significant improvement was only observed in NBG. In conclusion, while both techniques can be used as effective training methods to improve WM, the dual n-back task training method, perhaps has a more prominent transfer effect than that of method of loci.

## Introduction

Working memory (WM) facilitates the process of temporary storage and manipulation of the information necessary for most higher level cognitive tasks, such as learning, reasoning, and comprehension^1–4^. Although WM plays an indispensable role for humans in aspects of daily life, academic performance and work, its capacity displays limitation. Moreover, deficits in WM are usually associated with several neuropsychiatric disorders, for instance, cognitive impairment^5^, Parkinson’s disease (PD)^6,7^, attention-deficit hyperactivity disorder (ADHD)^2,8,9^, Alzheimer’s disease (AD)^7,10,11^ and schizophrenia^11,12^. Encouragingly, increasing evidence on brain plasticity demonstrate that memory performance improvement can be achieved via memory training^2,13–17^. The two major memory training approaches are: process-based memory training^18–22^ and strategy-based memory training^23–25^.

Process-based memory training mostly focuses on enhancing capacities related to operations such as processing speed and executive functions^7,15,26,27^. The dual n-back task, which is one of the most frequently utilized experimental paradigms for process-based training^19,28,29^, involves simultaneous serial presentation of auditory and visual stimuli that requires participants to make a specific response on either the identity or location when the current stimulus matches the one presented n trials back^18–20,28^. Numerous studies have linked n-back training to improvement of WM capacity^18,19,26,29–31^, near transfer to performance of structurally similar WM tasks^22,30,32^, and even far transfer to fluid intelligence^18,19,22,31,32^. However, some inconsistent results indicate that failures of positive transfer of training to other unrelated WM tasks still exist^33–35^. Therefore, more evidence is needed to support n-back training induced performance effect reliable transference to other cognitive tasks.

Strategy-based memory training on the other hand refers to improving memory performance by applying mnemonic strategies which contributes to information encoding and retrieval^13,36^. Multiple types of strategies such as method of loci, rehearsal, imagery, associations, categorization are regarded as effective mnemonic strategies^37^. Although the method of loci, also called memory palace technique^23,24,38^, is one of the most prevalent strategies^24,25^. The successful implementation of the method of loci strategy depends on the creation of a familiar visuo-spatial mnemonic environment filled with landmarks, imagined navigation whereby location is paired with one or more to-be-remembered items when encoding, and “mentally walking” through the created environment again to sequentially retrieve those items^24,25,37^. Despite the considerable amount of literature reporting the benefits of the aforementioned method^38–46^, a number of studies demonstrate its limitation in transfer effect^15,37,47,48^.

In the behavioral WM training researches, transfer of training gains to trained or untrained tasks has always been the central concern. The n-back task is often used for WM training as noted before, and has also been widely considered as an updating measure of WM capacity^22,49–51^. Whereas, the words memory task is usually taken as an evaluation of mnemonic strategy training^25,52^. These two tasks, therefore, are separately considered as a measure of task-specific (trained task) transfer according to the training groups in our study. Gain in WM capacity is what all WM training techniques want to achieve. The digit-span has often been regarded as non-trained measure of short-term memory/WM capacity^9,22,53–55^. Furthermore, the visuo-spatial change detection task, which involves the remembering of spatial locations of objects^56^, is formally similar with n-back task and method of loci on memory environment of spatial domain. Therefore, change detection task can also be used as an assessment of transfer gains. In our study, we adopted these four cognitive tasks as measures of training outcomes.

As mentioned above, both processed-based memory training (n-back task) and strategy-based memory training (method of loci) have been frequently applied in interventions aimed at improving WM performance. However, few studies have compared directly the effect between the two training techniques^25,48^, and in addition, the transfer effect of each technique is still controversial. Therefore, our study recruited two training groups, N-back group (NBG) and Memory Palace group (MPG), separately training with n-back task and the method of loci, and a blank control group (BCG) who received no training as a contrast. All groups were tested on two trained tasks and two untrained tasks at both pre-test and post-test. The aims of the study were to further examine whether both training techniques improve the performance of directly trained task as suggested by previous studies (aim 1) and whether training transfer can be generalized to non-trained tasks involving different types of WM abilities (aim 2), and to compare the effect between training groups (aim 3). Based on evidence mentioned before, we hypothesized that both training groups may gain great improvement on trained tasks and untrained tasks. Generally, we assumed that n-back training may have a more significant transfer effect than that of method of loci on the untrained tasks.

## Results

### Training performance

**Figure 1:**
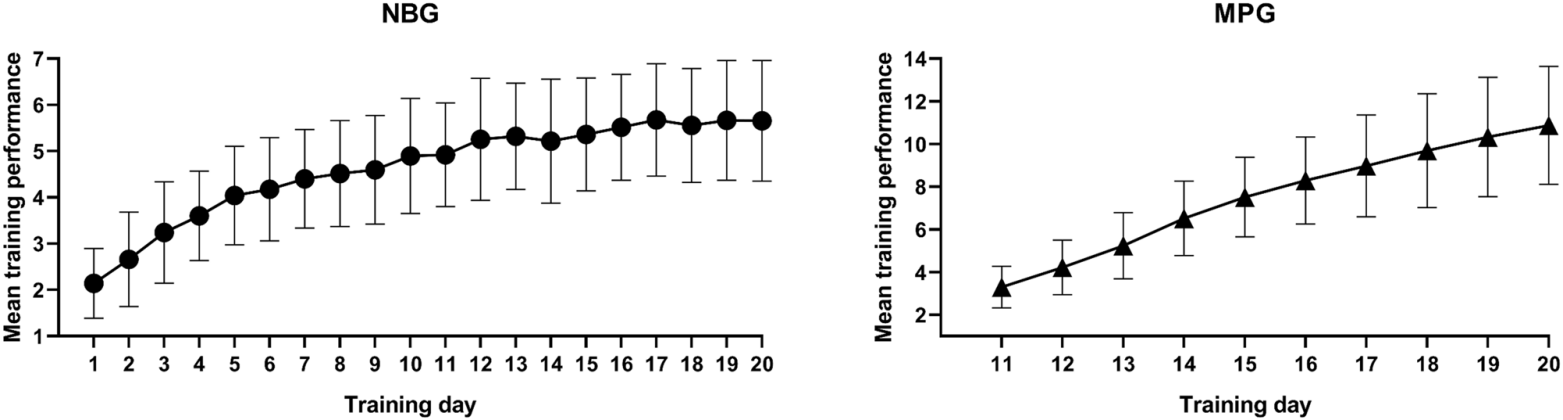
Performance improvement over training days. These two graphs separately show the linear improvement in average training performance across the training days for each training group. Error bars indicates SD. *NBG* n-back group, *MPG* memory palace group.

Both NBG and MPG showed a linear improvement across memory training on their respective training task (Fig. 1). The average daily maximum performance of NBG improved from n = 2.21 (SD = 0.798) at day 1 to n = 5.71 (SD = 1.336) at day 20 (t_(47)_ = 18.358, *p* < 0.001). In the MPG, the mean daily maximum performance of words memory also increased gradually from the beginning of memory test (day 11) (M = 3.34, SD = 0.984) to the end (day 20) (M = 10.87, SD = 2.763) (t_(46)_ = 19.171, *p* < 0.001).

### Training effects on trained tasks

#### Dual n-back task

**Figure 2:**
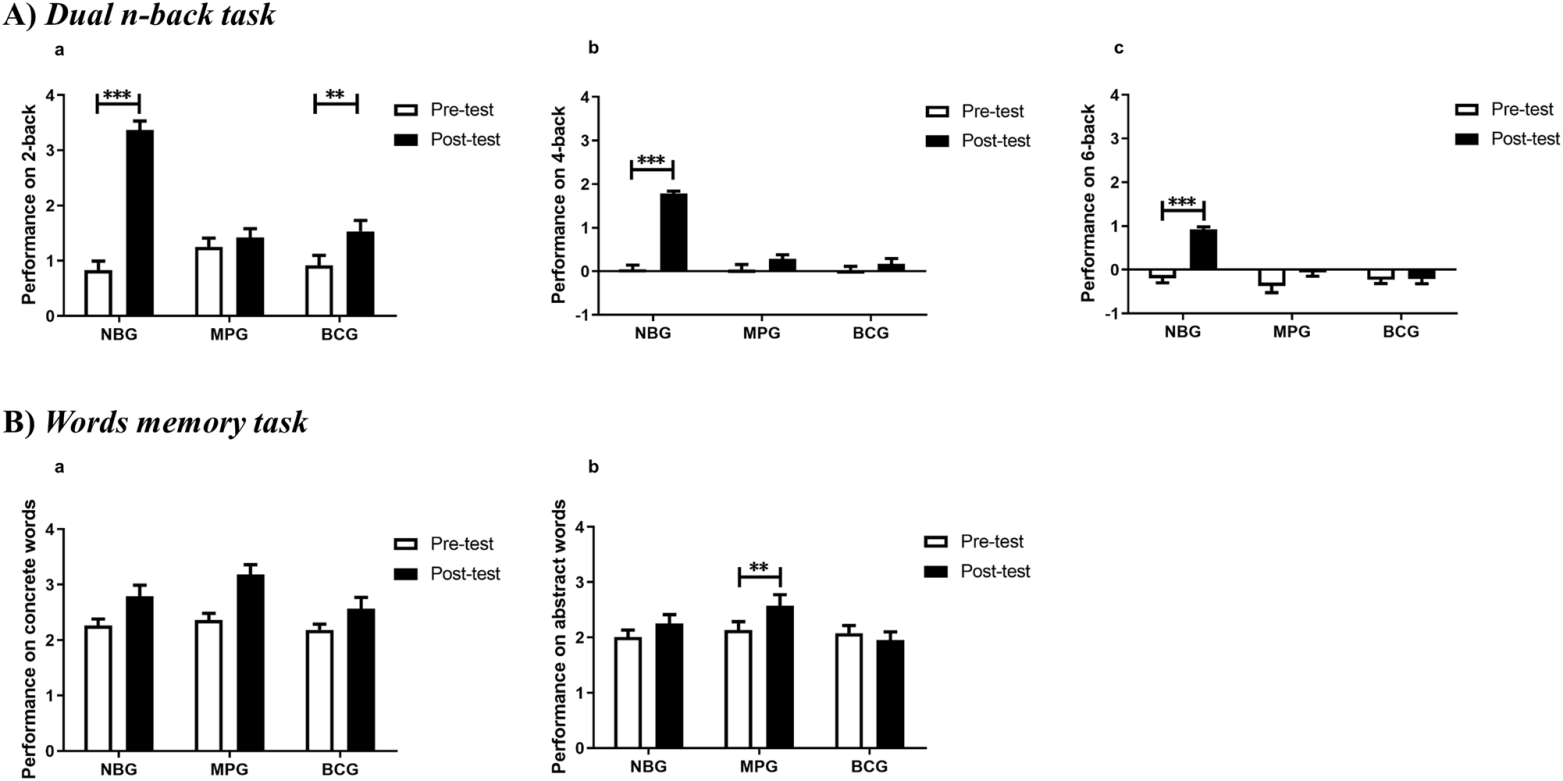
Performance on trained tasks for each group at pre-test and post-test. (**A**) Dual n-back task. Graph a reports performance on 2-back, graph b on 4-back, and graph c on 6-back. (**B**) Words memory task. Graph a displays performance on concrete words memory, and Graph b on abstract words memory. Error bars indicate SEM. *NBG* n-back group, *MPG* memory palace group, *BCG* blank control group.

There were no significant group differences on d prime of any load at pre-test (2-back: F_(2,136)_ = 1.774, *p* = 0.174; 4-back: F_(2,136)_ = 0.008, *p* = 0.992; 6-back: F_(2,136)_ = 0.643, *p* = 0.527). The three-way (2 time points × 3 groups × 3 conditions) ANOVA analysis revealed main effects of time (F_(1,136)_ = 152.626, *p* < 0.001, ${\eta }_{\mathrm{\rm P}}^{2}$=0.529), group (F_(2,136)_ = 37.712, *p* < 0.001, ${\eta }_{\mathrm{\rm P}}^{2}$=0.357), and condition (F_(2,272)_ = 271.396, *p* < 0.001, ${\eta }_{\mathrm{\rm P}}^{2}$=0.666). It also revealed interactions of time × group (F_(2,136)_ = 71.460, *p* < 0.001, ${\eta }_{\mathrm{\rm P}}^{2}$=0.512), time × condition (F_(2,272)_ = 12.858, *p* < 0.001, ${\eta }_{\mathrm{\rm P}}^{2}$=0.086), and time × condition × group (F_(4,272)_ = 6.881, *p* < 0.001, ${\eta }_{\mathrm{\rm P}}^{2}$=0.092). No significant interaction for condition × group emerged (*p* = 0.487). To further demonstrate training effect, two-way (2 time points × 3 groups) ANOVA was separately applied for d prime comparisons on each condition (Fig. 2A; Table 1). For 2-back, we found significant main effects of time (F_(1,136)_ = 81.026, *p* < 0.001, ${\eta }_{\mathrm{\rm P}}^{2}$=0.373) and group (F_(2,136)_ = 12.796, *p* < 0.001, ${\eta }_{\mathrm{\rm P}}^{2}$=0.158), as well as a highly significant group × time interaction (F_(2,136)_ = 36.435, *p* < 0.001, ${\eta }_{\mathrm{\rm P}}^{2}$=0.349). Further post-hoc t-tests revealed that both NBG (*p* < 0.001) and BCG (*p* = 0.003) resulted in a significant improvement in 2-back from pre-test to post-test, but this was not the case for MPG (*p* = 0.397).

**Table 1:** Training effects on cognitive tasks.

	NBG		MPG		BCG		F	*P*	${\eta }_{p}^{2}$
	Pre-test	Post-test	Pre-test	Post-test	Pre-test	Post-test			
	M (SEM)	M (SEM)	M (SEM)	M (SEM)	M (SEM)	M (SEM)			
**Dual n-back task**	n = 50		n = 47		n = 42				
2-back (d′)	0.83 (0.17)	3.37 (0.16)	1.25 (0.16)	1.42 (0.16)	0.91 (0.18)	1.53 (0.20)	36.435	< 0.001	0.349
4-back (d′)	0.04 (0.10)	1.78 (0.06)	0.04 (0.12)	0.29 (0.09)	0.02 (0.09)	0.17 (0.12)	46.983	< 0.001	0.409
6-back (d′)	− 0.19 (0.10)	0.92 (0.06)	− 0.37 (0.15)	− 0.06 (0.09)	− 0.23 (0.09)	− 0.21 (0.11)	17.231	< 0.001	0.202
**Words memory task**	n = 49		n = 50		n = 48				
Concrete words (d′)	2.26 (0.12)	2.79 (0.20)	2.37 (0.12)	3.18 (0.18)	2.18 (0.10)	2.57 (0.20)	1.305	0.274	0.018
Abstract words (d′)	2.01 (0.13)	2.26 (0.16)	2.14 (0.15)	2.58 (0.20)	2.08 (0.14)	1.95 (0.15)	3.434	0.035	0.046
**Digit-span task**	n = 50		n = 50		n = 48				
Digit-span	9.30 (0.18)	10.14 (0.18)	9.62 (0.16)	9.98 (0.20)	9.50 (0.19)	9.67 (0.19)	4.193	0.017	0.055
**Change detection task**	n = 47		n = 49		n = 46				
Load 2 (RT)	750.46 (21.35)	678.01 (18.41)	712.77 (21.94)	663.95 (16.83)	740.21 (22.30)	707.23 (18.19)	1.607	0.204	0.023
Load 4 (RT)	777.97 (20.44)	700.84 (16.76)	728.94 (21.35)	695.88 (19.18)	760.25 (21.02)	730.91 (17.26)	3.046	0.051	0.042
Load 6 (RT)	818.63 (20.60)	740.40 (19.35)	761.26 (21.30)	726.37 (18.70)	790.50 (20.99)	772.56 (19.43)	3.962	0.021	0.054

The statistic parameters in the table indicate interactions of group × time.

*d′* d prime, *NBG* n-back group, *MPG* memory palace group, *BCG* blank control group.

For 4-back, we also found main effects of time (F_(1,136)_ = 87.895, *p* < 0.001, ${\eta }_{\mathrm{\rm P}}^{2}$=0.393) and group (F_(2,136)_ = 40.466, *p* < 0.001, ${\eta }_{\mathrm{\rm P}}^{2}$=0.373), and an interaction as well (F_(2,136)_ = 46.983, *p* < 0.001, ${\eta }_{\mathrm{\rm P}}^{2}$=0.409). Post-hoc t-tests showed that only NBG gained in d prime after training (*p* < 0.001), but no significant increase was found in the MPG (*p* = 0.092) or BCG (*p* = 0.240).

For 6-back, we still observed main effects of time (F_(1,136)_ = 35.908, *p* < 0.001, ${\eta }_{\mathrm{\rm P}}^{2}$=0.209) and group (F_(2,136)_ = 19.806, *p* < 0.001, ${\eta }_{\mathrm{\rm P}}^{2}$=0.226), and an interaction (F_(2,136)_ = 17.231, *p* < 0.001, ${\eta }_{\mathrm{\rm P}}^{2}$=0.202). Further t-tests demonstrated that only a significant enhancement was observed in NBG (*p* < 0.001) rather than the MPG (*p* = 0.063) and BCG (*p* = 0.919) after training.

#### Words memory task

The three groups did not show significant differences on performance of concrete or abstract words memory at the beginning (concrete: F_(2,144)_ = 0.645, *p* = 0.526; abstract: F_(2,144)_ = 0.209, *p* = 0.812). The three-way (2 time points × 3 groups × 2 word categories ) ANOVA analysis revealed main effects of time (F_(1,144)_ = 21.739, *p* < 0.001, ${\eta }_{\mathrm{\rm P}}^{2}$=0.131), and word category (F_(1,144)_ = 36.689, *p* < 0.001, ${\eta }_{\mathrm{\rm P}}^{2}$=0.203); and revealed interactions of time × group (F_(2,144)_ = 3.075, *p* = 0.049, ${\eta }_{\mathrm{\rm P}}^{2}$=0.041), and time × word category (F_(1,144)_ = 10.931, *p* = 0.001, ${\eta }_{\mathrm{\rm P}}^{2}$=0.071). No other significant main effects or interactions emerged from this analysis (all other *p* > 0.08). To further explore training effect, two-way (2 time points × 3 groups) ANOVA was separately applied for d prime comparisons on each word category (Fig. 2B; Table 1). For concrete words, repeated ANOVA analysis revealed a significant main effect of time (F_(1,144)_ = 27.038, *p* < 0.001, ${\eta }_{\mathrm{\rm P}}^{2}$=0.158), no main effect of group (*p* = 0.085) nor an interaction (*p* = 0.274). For abstract words, there was a significant main effect of time (F_(1,144)_ = 4.422, *p* = 0.037, ${\eta }_{\mathrm{\rm P}}^{2}$=0.030) and an interaction between time point and group (F_(2,144)_ = 3.434, *p* = 0.035, ${\eta }_{\mathrm{\rm P}}^{2}$=0.046), but no main effect of group (*p* = 0.193). Further post-hoc t-tests revealed that only MPG (*p* = 0.008) achieved a significant improvement, but this was not true for NBG (*p* = 0.102) and BCG (*p* = 0.422).

### Training effects on untrained tasks

#### Digit-span task

**Figure 3:**
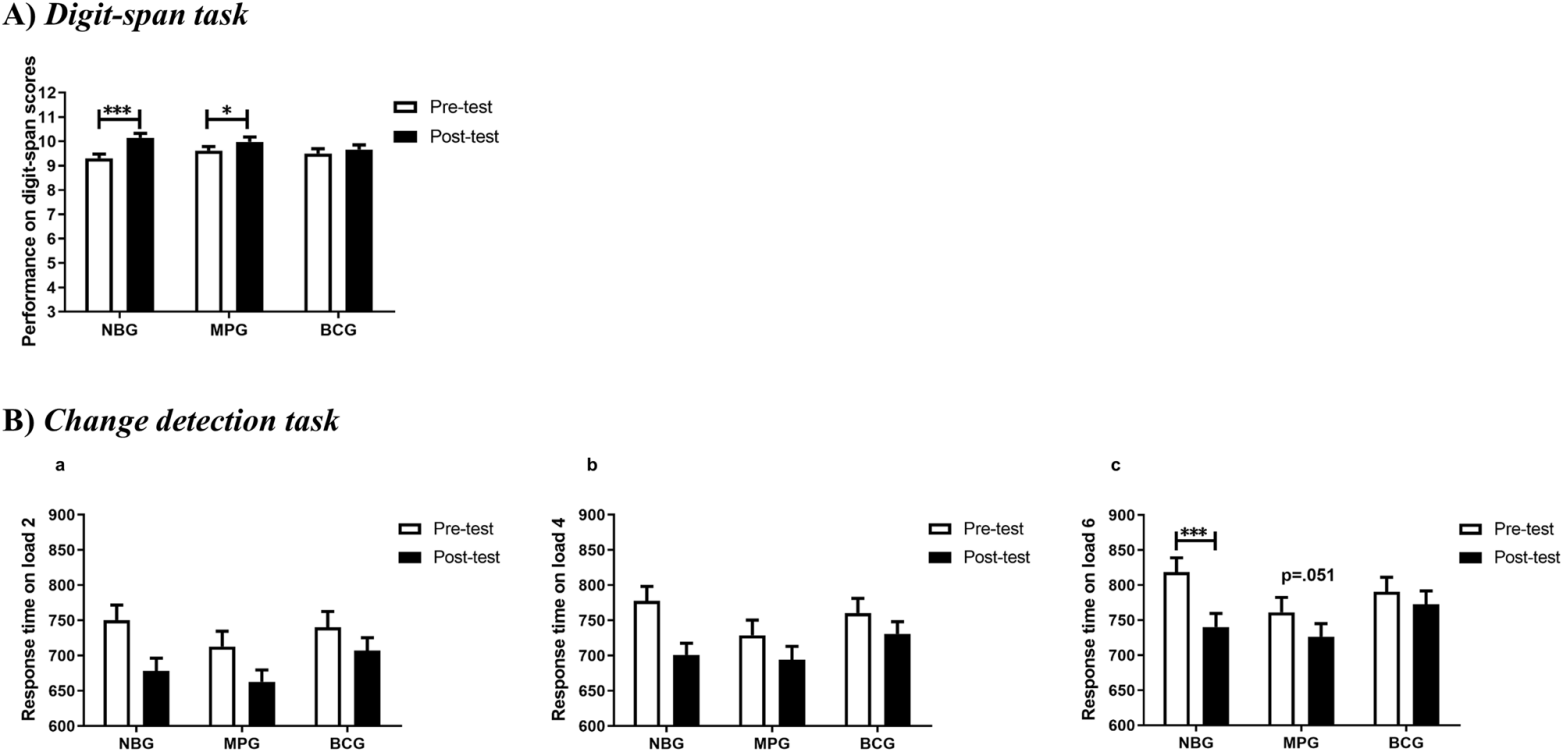
Performance on untrained tasks for each group at pre-test and post-test. (**A**) Digit-span task. The figure represents performance on digit-span scores. (**B**) Change detection task. Graph a depicts response time on load 2, graph b on load 4, and graph c on load 6. Error bars indicate SEM. *NBG* n-back group, *MPG* memory palace group, *BCG* blank control group.

Significant group differences were not observed on digit-span scores before training (F_(2,145)_ = 0.830, *p* = 0.438). A repeated ANOVA analysis was further recruited to examine the training effect on digit-span scores (Fig. 3A; Table 1). As predicted, a significant main effect of time (F_(1,145)_ = 21.727, *p* < 0.001, ${\eta }_{\mathrm{\rm P}}^{2}$=0.130) and interaction (F_(2,145)_ = 4.193, *p* = 0.017, ${\eta }_{\mathrm{\rm P}}^{2}$=0.055) were observed. Post-hoc t-tests revealed an increased performance in NBG (*p* < 0.001) and MPG (*p* = 0.038), and no significant increase in BCG (*p* = 0.315).

#### Change detection task

Significant group differences in response time (RT) of location repeat condition did not appear in the pre-test (load 2: F_(2,139)_ = 0.808, *p* = 0.448; load 4: F_(2,139)_ = 1.427, *p* = 0.244; load 6: F_(2,139)_ = 1.895, *p* = 0.154). The three-way (2 time points × 3 groups × 3 loads) ANOVA analysis revealed main effects of time (F_(1,139)_ = 32.223, *p* < 0.001, ${\eta }_{\mathrm{\rm P}}^{2}$=0.188), and load (F_(2,278)_ = 156.385, *p* < 0.001, ${\eta }_{\mathrm{\rm P}}^{2}$=0.529); and revealed an interaction of time × group (F_(2,139)_ = 3.129, *p* = 0.047, ${\eta }_{\mathrm{\rm P}}^{2}$=0.043). No other significant main effects or interactions emerged from this analysis (all other *p* > 0.3). To further establish the influence of training on RT, two-way ANOVA analysis was separately performed on each load (Fig. 3B; Table 1). For load 2, we found a main effect of time (F_(1,139)_ = 32.785, *p* < 0.001, ${\eta }_{\mathrm{\rm P}}^{2}$=0.191), and neither a main effect of group (*p* = 0.369) nor interaction (*p* = 0.204). For load 4, there was also only main effect of time (F_(1,139)_ = 28.130, *p* < 0.001, ${\eta }_{\mathrm{\rm P}}^{2}$=0.168), and no main effect of group (*p* = 0.377) or interaction (*p* = 0.051). For load 6, we observed a main effect of time (F_(1,139)_ = 23.745, *p* < 0.001, ${\eta }_{\mathrm{\rm P}}^{2}$=0.146) and a significant interaction (F_(2,139)_ = 3.962, *p* = 0.021, ${\eta }_{\mathrm{\rm P}}^{2}$=0.054), however, we did not find a main effect of group (*p* = 0.267). Further t-tests exhibited a statistically significant decrease of RT in NBG (*p* < 0.001), but this was not the case for MPG (*p* = 0.051) and BCG (*p* = 0.204).

Significant group differences in accuracy (ACC) of location repeat condition did not show in the pre-test (load 2: F_(2,139)_ = 0.265, *p* = 0.767; load 4: F_(2,139)_ = 0.273, *p* = 0.762; load 6: F_(2,139)_ = 0.983, *p* = 0.377). The three-way (2 time points × 3 groups × 3 loads) ANOVA analysis revealed main effects of time (F_(1,139)_ = 6.216, *p* = 0.014, ${\eta }_{\mathrm{\rm P}}^{2}$=0.043), and load (F_(2,278)_ = 4.978, *p* = 0.013, ${\eta }_{\mathrm{\rm P}}^{2}$=0.035). No other significant main effects or interactions emerged from this analysis (all other *p* > 0.2).

## Discussion

In the present study, we applied memory training techniques with n-back task for NBG and method of loci for MPG to improve WM performance. Two trained tasks, dual n-back task and words memory task, and other two non-trained tasks, digit-span task and change detection task, were measured at both pre-test and post-test. Both training groups resulted in greater training improvement, with NBG displaying significant performance improvement of n-back task, and MPG in words memory task. For untrained tasks, both training groups yielded significant transfer effect to digit-span scores. However, for change detection task, only NBG showed significant improvement on response speed, while MPG did not show significant enhancement.

In accordance with previous evidence suggesting that n-back training can increase WM capacity and task performance^18,19,26,29–31^, our study also observed great improvement on performance of each day training as well as on performance of dual n-back task after training in NBG. Moreover, the significant performance improvement on dual n-back task was observed uniquely in NBG rather than MPG or BCG for higher task loads (4-back and 6-back). For 2-back task load, significant improvement in performance was realized in both NBG and BCG. Perhaps this may be due to the learning effect of easy tasks, such as n-back task with lower loads in BCG. However, there was no significant effect on performance in 2-back task load in MPG, implying that there was no learning effect. A possible explanation for this is that perhaps the memory process of squares and consonants was intervened by the utilization of method of loci, which is successful in enhancing episodic memory with items such as words and names^36,48^. Considering task difficulty of 6-back in n-back task, 2- to 4-back trained task loads may be more suitable to obtain more accurate measures of the effect of different training techniques.

For words memory task, training effect was found uniquely in MPG on performance of abstract words rather than concrete words. On one hand, it is well known that the processing of abstract words is much more difficult than that of concrete words^57–63^. On the other hand, the utility of method of loci has been reported to facilitate the transformation of abstract information into concrete information^25^, which can be further processed more easily by memory-related neural system. It is, therefore, not surprising that the WM capacity for abstract words increased in response to training of method of loci. Additionally, all groups resulted in improvement on concrete words memory in post-test as compared to those in pre-test. The observed performance increase in concrete words in all groups could also be attribute to learning effect, because, in comparison to abstract words, concrete words indeed need relatively limited available contextual information to encode^57–60^.

For digit-span task, significant improvements were observed in both NBG and MPG, which is consistent with our initial expectation. Forward digit-span test is often used to assess near transfer effects, i.e. short-term memory or WM capacity, in WM training^9,21,22,53–55^. As our results demonstrate, both n-back training and method of loci could effectively promote capacity of short-term memory for digits. Concerning short-term memory, it is particularly worth mentioning that temporary storage is essential for the implementation of dual n-back task, and perhaps, the short-term memory limited capacity can be increased either on the verbal or spatial domain through longtime training^26^. Most studies have documented significant transfer effect of n-back training gains to digit-span scores^18,30,54^. Similarly, our study observed this improvement not only in NBG but also in MPG. In digit-span task, the to-be-remembered items (digits) are presented in a continuous stream, while the implementation of method of loci also focuses on sequence memory, with items put in sequential landmarks when encoding as well as when retrieving them in order during recall phase. Therefore, the significant improvement on short-term memory capacity for digits in MPG may benefit from the repeated training on similar form of sequential memory.

As for the processing speed improvement in change detection task, we only observed a significant improvement on higher load in NBG, but not in MPG. This was somewhat unexpected, given the initially similar spatial memory environment with the method of loci, and hence contrary to our hypothesis. Some studies have reported that in order to acquire substantial transfer effect, the training paradigm and untrained task must share relevant information-processing components and be involved in similar neural substrate^64^. Coincidentally, the central mechanism of process-based training technique aims at producing more stable effects in functions that engage a common neural circuitry^65^. Previous evidence demonstrate that the performance of n-back task recruits the fronto-parietal executive control network^28,48,66,67^, the same neural network also involved in change detection task^56^. More so, recent meta-analytical evidence further indicates that more substantial transfer may only occur when the trained task and transfer task share the same task paradigm^22,68^. In the n-back task, the to-be-remembered items are presented in a continuous stream, and participants must make quick responses to repeated targets according to the task loads. RT represents processing speed, which is one of processing capacities that process-based training hope to enhance^15,27^. Training may increase the efficiency of this process by accelerating responses to repeated target trials. So training-induced improvement in potential ability may be transferred to other ability to detect repeated objects in change detection task that involve similar task demand with n-back task. Demonstrably, these two kind of tasks have much in common either on task paradigm or neural substrate they are involved in.

Conversely, the method of loci has most often been associated with the episodic memory domain, where performance is enhanced by facilitating encoding and retrieval of information of unrelated word pairs^48^. Therefore, the lack of positive transfer to reduced RT may be due to the specificity of this mnemonic strategy with limited applicability to other ability-related domains including processing speed. Rebok et al.^47^ also summarized in their paper that training outcomes are highly specific to the ability of mnemonic strategies that have been trained. Moreover, the limitation effect of mnemonic strategy training has been proved to be linked to age with less training-induced changes in older adults than in younger ones^46,69^. Precisely, the elderly adults usually encounter great difficulty in applying mnemonic strategy to their daily lives^15^.

Generally, our results are consistent with our expectation that n-back training produces relatively prominent transfer effects compared to method of loci on the untrained tasks. However, these findings on one hand, may be partly due to the possibility that the two untrained tasks, one selected as the measure of WM capacity and the other as the measure of processing speed, are more involved in the common processing mechanism, which happens to be the central component that the process-based WM training focuses on. More so, in our study, the two untrained measure tasks seem to be more similar to dual n-back task on paradigm form. If the preceding arguments are valid, this may also reveal one major limitation of our study, that the measures of outcomes might be biased on a single training technique. Therefore, the future studies should bring into consideration the possible influence of biased measure task so as to clearly ascertain whether the training techniques are successfully improving the WM performance. More feasibly, comprehensive assessments covering both training domains should be employed optimally to investigate the training effects of the two memory techniques in the future. On the other hand, mnemonic strategy of method of loci may indeed have a limitation transferring to other situations sharing relevant ability. Although previous findings on memory training in older adults suggest that strategy-based training may be less effective as compared to process-based training technique^15^. Therefore, more evidence including broader age domains may be needed before a general conclusion of the effectiveness between these two training techniques is drawn. Importantly, in the present study, only two most frequently applied training techniques were selected in order to comprehensively compare the effect of the process-based training to that of the strategy-based training. Hence, we also recommend that a variety of training methods, deriving from both process-based and strategy-based training techniques, should be recruited to training experiments in future studies.

In conclusion, the current study recruited three group of participants to investigate memory training effect, and both training groups resulted in great improvement in WM capacity. In particular, n-back training yielded a more prominent transfer of training gains to untrained tasks than training of method of loci. Therefore, it may be recommendable to adopt process-based training for participants with multiple cognitive deficits or persons requiring enhancement of cognitive functions.

## Methods

### Participants

To determine the sample size, we considered a medium effect size (Cohen’s d = 0.52) suggested by Melby-Lervåg and Hulme^70^ in their meta-analytic study on the effectiveness of working memory training. Notably, the Cohen’s d = 0.52 equates to Cohen’s f = 0.26, according to online conversion calculator^71^ (*Psychmetrica,* https://www.psychometrica.de/effect_size.html). Then we performed a power analysis using G*Power^72^, with alpha = 0.05, power = 0.95, and effect size = 0.26 in ANOVA (between-within interaction). From our analysis, the required number of participants was 63. However, to further ascertain the appropriate sample size, we reviewed literature on the related work. Precisely, a meta-analytic study by Salmi et al.^14^ indicate that most working memory training studies recruit between 5 and 50 participants per training group. Therefore, we chose a threshold of 50 participants per group in our study.

**Figure 4:**
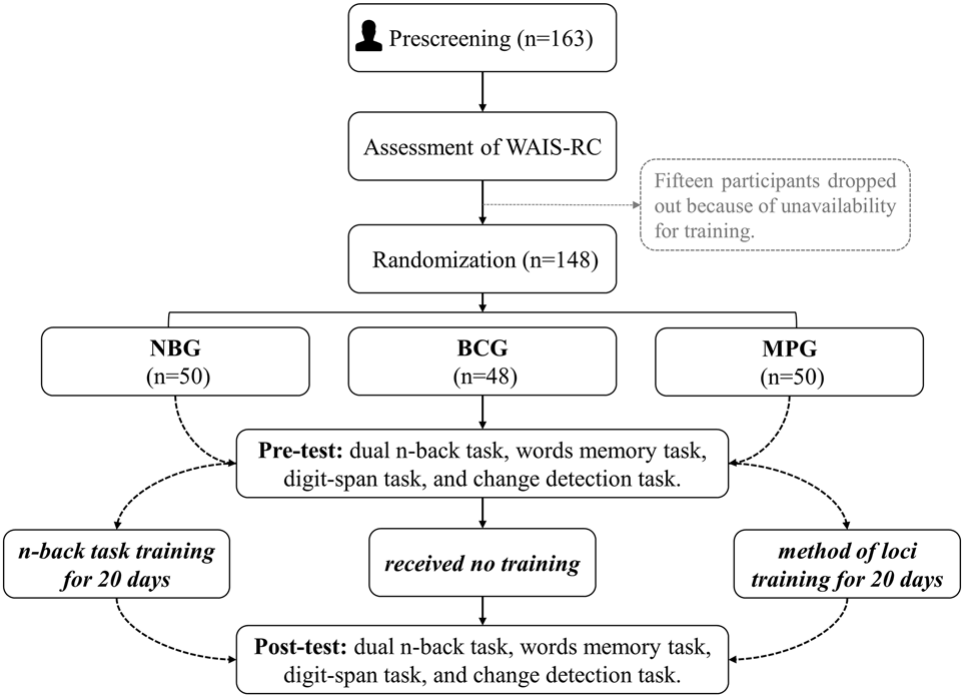
The flow of the study. *WAIS-RC* Wechsler Adult Intelligence Scale-Revised in China. *NBG* n-back group, *MPG* memory palace group, *BCG* blank control group.

Accordingly, one hundred and sixty-three (163), healthy right-handed participants in total (75 female; mean age: 21.22; age range: 18–26 years) were initially recruited from the University of Electronic Science and Technology of China (UESTC). All participants reported normal (or corrected-to-normal) vision, had no history of mental disorders, and gave written informed consent. After undertaking the test of Wechsler Adult Intelligence Scale-Revised in China (WAIS-RC)^73^, and the exclusion of fifteen participants who reported that they may not have enough time to complete training every day, the remaining participants were assigned randomly and evenly across three different groups: 50 (22 female; mean age 21.06 ± 1.932 SD years), 50 (25 female; mean age 21.1 ± 1.961 SD years) and 48 (24 female; mean age 21.38 ± 1.566 SD years) for NBG, MPG, and BCG respectively. Figure 4 shows the flow of the study. Three groups did not show significant difference in demographic characteristics consisting of gender, age and scores of WAIS-RC (all *p* > 0.2) (Table 2). This experiment was approved by the local committee for the Protection of Human Subjects of the UESTC and was conducted in accordance with the declaration of Helsinki.

**Table 2:** Demographic characteristics.

	NBG	MPG	BCG	*P*
	(n = 50)	(n = 50)	(n = 48)	
	M (SD)	M (SD)	M (SD)	
Female/male	22/28	25/25	24/24	0.788
Age (years)	21.06 (1.932)	21.1 (1.961)	21.38 (1.566)	0.654
WAIS-RC	126.6 (6.168)	128.44 (6.926)	126.63 (6.765)	0.287

*WAIS-RC* Wechsler Adult Intelligence Scale-Revised in China, *NBG* n-back group, *MPG* memory palace group, *BCG* blank control group.

### Procedure

All participants were tested with four cognitive tasks (described below) lasting for about two hours at both pre-test and post-test. The training session lasted for 20 days, with four consecutive weeks and five days per week. The training groups spent 30 min each day in the laboratory to complete the corresponding working memory training task (described below), whereas the BCG did not get any training during the 20 days. All participants in both training groups completed more than the required 80% of the training days, with 96% participants completing 19-day and 94% completing 20-day training. All training processes were under supervision of experimenters, who took charge of solving problems of experimental procedures, checking training data every day, and informing participants to make up for the missed training time if necessary.

### Memory training paradigms

#### The dual n-back training task

We used a classic WM training paradigm employed by Jaeggi et al.^18^, see Supplementary Material, Fig. S1A. Two kind of stimuli were displayed in the experiment, with squares (visual stimuli) presented sequentially at eight different locations of the computer screen and consonants (auditory stimuli, eight in total), one at a time in sequence, presented simultaneously through headsets. When one of the presented stimuli matched the one presented n positions back in the sequence, a response should be made, with pressing letter “A” on the keyboard for visual targets and letter “L” for auditory targets. Six visual and six auditory targets appeared randomly in each block, with only two appearing simultaneously in both streams of stimuli. So ten targets in total were presented in each block, consisting of 20 + n trials, where n represented the level of difficulty of current block. The value of n of the next block changed adaptively based on individual performance of the current block.

#### The method of loci

We applied the paradigm of method of loci^25^^.^as a strategy-based training technique. This training process included two sessions with 10 days each. The first 10 days were used to introduce the method of loci to participants, and guide participants to remember loci routes with several landmarks, within which they were trained to memorize random words associated with landmarks. Five loci routes of samples were remembered and applied skillfully, and a new loci route based on individual experience that was more suitable for oneself to memorize words was established to be used in the subsequent stage. The second session was to memorize random words using an adaptive vocabulary memory software developed by our laboratory. The initial memory level started with 5 random words, and the duration of memory time limited to 90 s. Each additional level was increased by 5 words, and correspondingly the memory time increased by 90 s, and so on. In the recall stage, participants were required to type the words they had just remembered into the test box in sequence, separated by spaces. If all words entered were absolutely correct and their sequence accurate, the next level began, otherwise the participants continued with the current level. The program recorded automatically the highest daily level achieved by each subject. More than 5000 nouns with the same word frequency selected from online corpus (http://corpus.zhonghuayuwen.org/Resources.aspx) were used in the experiment.

### Trained memory tasks

#### Dual n-back task

For this task, we used the same material as the dual n-back training task. The only difference is that the levels of load were fixed to three conditions, i.e. n = 2, 4 and 6. There were three sessions presented in the task, each comprising of three blocks of different loads with sequence in 2–4–6 back, 4–6–2 back or 6–2–4 back.

#### Words memory task

This paradigm was used to test the performance of words memory^25,52^. The task consisted of two sessions, words encoding session and words recognition session, see Supplementary Material, Fig. S1B. In the first session, a random list of 72 two-character words, including 36 concrete words and 36 abstract words, was presented at a rate of 2 s per word. After continual presentation of 6 words, 20 s were given to participants to recall words presented before or take a temporal rest. Five minutes later after the end of the words memory session, a list of 144 words, consisting of 72 words presented before and 72 new words (half concrete, half abstract words), was presented to participants in a random order. During words recognition session, participants were required to indicate whether the presented word was old or new as soon as possible by pressing one of two buttons on the standard keyboard.

### Non-trained memory tasks

#### Digit-span task

The digit-span task (forward version) came from the WAIS-RC^73^. The length of digit-span was 3–12 bit. Participants were asked to verbally repeat lists of numbers in the same order at the end of each auditory presentation. If the participant failed twice at the same number list, the last number list repeated successfully will be recorded as final score.

#### Change detection task

The paradigm was modified from previous design^56^, see Supplementary Material, Fig. S1C. The disks of 9 colors without repetition were selected as attended items, which might appear randomly in 10 possible locations on an invisible 2 × 5 matrix in both left and right visual fields, with 2, 4 or 6 disks per hemi-field. A memory array consisting of the same number of disks in both visual fields were presented to participants after a prior presentation of an arrow, which instructed participants to memorize the items in the corresponding visual field. After a delay of 1600 ms, the test array were presented, where participants were asked to make a judgement on whether the locations of the disks in the attended hemi-field were the same or different from those in the memory array regardless of object colors. Location repeat condition meant none change of items locations from memory array to test array, while location change condition indicated that one item changed its location in the test array.

### Data analysis

Due to problems such as program errors and data transmission during the experiment, some data was damaged and rendered unusable. Therefore, not every cognitive task had 148 pieces of complete data: 139 data for dual n-back task, 147 data for words memory task, 148 data for digit-span task and 142 data for change detection task.

All data were analyzed with SPSS Statistics version 21.0 (Inc., an IBM Company). To compare the baseline performance on cognitive tasks among three groups, we employed one-way analyses of variance (ANOVA). To elucidate training efficacy, three-way and two-way repeated measures ANOVA were conducted separately in each cognitive task with group (NBG vs. MPG vs. BCG) as a between-subjects factor and time (pre-test vs. post-test) as a within-subjects factor. For all ANOVA analyses, Greenhouse–Geisser corrections were used for non-sphericity data as needed and Bonferroni corrections were applied for post-hoc multiple comparisons. Partial eta square (${\eta }_{p}^{2}$) was treated as effect size and *p* < 0.05 was considered as statistically significant.

## Supplementary Information

Supplementary Figure S1.

## References

[1] Cowan N (2017). The many faces of working memory and short-term storage. Psychon. Bull. Rev..

[2] Constantinidis C, Klingberg T (2016). The neuroscience of working memory capacity and training. Nat. Rev. Neurosci..

[3] Teixeira-Santos AC (2019). Reviewing working memory training gains in healthy older adults: A meta-analytic review of transfer for cognitive outcomes. Neurosci. Biobehav. Rev..

[4] Baddeley A (2003). Working memory: Looking back and looking forward. Nat. Rev. Neurosci..

[5] LópezZunini RA (2016). Event-related potentials elicited during working memory are altered in mild cognitive impairment. Int. J. Psychophysiol..

[6] Ophey A (2020). Effects of working memory training in patients with Parkinson’s disease without cognitive impairment: A randomized controlled trial. Park. Relat. Disord..

[7] Jiang Y, Abiri R, Zhao X (2017). Tuning up the old brain with new tricks: Attention training via neurofeedback. Front. Aging Neurosci..

[8] Maehler C, Schuchardt K (2016). Working memory in children with specific learning disorders and/or attention deficits. Learn. Individ. Differ..

[9] Ackermann S, Halfon O, Fornari E, Urben S, Bader M (2018). Cognitive Working Memory Training (CWMT) in adolescents suffering from Attention-Deficit/Hyperactivity Disorder (ADHD): A controlled trial taking into account concomitant medication effects. Psychiatry Res..

[10] Hampstead BM, Sathian K, Bikson M, Stringer AY (2017). Combined mnemonic strategy training and high-definition transcranial direct current stimulation for memory deficits in mild cognitive impairment. Alzheimer’s Dement. Transl. Res. Clin. Interv..

[11] Gilmour G (2019). Relating constructs of attention and working memory to social withdrawal in Alzheimer’s disease and schizophrenia: issues regarding paradigm selection. Neurosci. Biobehav. Rev..

[12] Grot S (2017). Abnormal prefrontal and parietal activity linked to deficient active binding in working memory in schizophrenia. Schizophr. Res..

[13] Nguyen L, Murphy K, Andrews G (2019). Cognitive and neural plasticity in old age: A systematic review of evidence from executive functions cognitive training. Ageing Res. Rev..

[14] Salmi J, Nyberg L, Laine M (2018). Working memory training mostly engages general-purpose large-scale networks for learning. Neurosci. Biobehav. Rev..

[15] Karbach J, Verhaeghen P (2014). Making working memory work: A meta-analysis of executive-control and working memory training in older adults. Psychol. Sci..

[16] Hampstead BM, Stringer AY, Stilla RF, Sathian K (2020). Mnemonic strategy training increases neocortical activation in healthy older adults and patients with mild cognitive impairment. Int. J. Psychophysiol..

[17] Salminen T, Mårtensson J, Schubert T, Kühn S (2016). Increased integrity of white matter pathways after dual n-back training. Neuroimage.

[18] Jaeggi SM, Buschkuehl M, Jonides J, Perrig WJ (2008). Improving fluid intelligence with training on working memory. Proc. Natl. Acad. Sci..

[19] Au J (2015). Improving fluid intelligence with training on working memory: a meta-analysis. Psychon. Bull. Rev..

[20] Shipstead Z, Redick TS, Engle RW (2012). Is working memory training effective?. Psychol. Bull..

[21] Borella E, Carbone E, Pastore M, De Beni R, Carretti B (2017). Working memory training for healthy older adults: The role of individual characteristics in explaining short- and long-term gains. Front. Hum. Neurosci..

[22] Soveri A, Antfolk J, Karlsson L, Salo B, Laine M (2017). Working memory training revisited: A multi-level meta-analysis of n-back training studies. Psychon. Bull. Rev..

[23] Peeters A, Segundo-Ortin M (2019). Misplacing memories? An enactive approach to the virtual memory palace. Conscious. Cogn..

[24] Legge ELG, Madan CR, Ng ET, Caplan JB (2012). Building a memory palace in minutes: Equivalent memory performance using virtual versus conventional environments with the Method of Loci. Acta Psychol..

[25] Dresler M (2017). Mnemonic training reshapes brain networks to support superior memory. Neuron.

[26] Lilienthal L, Tamez E, Shelton JT, Myerson J, Hale S (2013). Dual n-back training increases the capacity of the focus of attention. Psychon. Bull. Rev..

[27] Nouchi R, Saito T, Nouchi H, Kawashima R (2016). Small acute benefits of 4 weeks processing speed training games on processing speed and inhibition performance and depressive mood in the healthy elderly people: Evidence from a randomized control trial. Front. Aging Neurosci..

[28] Owen AM, McMillan KM, Laird AR, Bullmore E (2005). N-back working memory paradigm: A meta-analysis of normative functional neuroimaging studies. Hum. Brain Mapp..

[29] Course-Choi J, Saville H, Derakshan N (2017). The effects of adaptive working memory training and mindfulness meditation training on processing efficiency and worry in high worriers. Behav. Res. Ther..

[30] Heinzel S (2014). Working memory load-dependent brain response predicts behavioral training gains in older adults. J. Neurosci..

[31] Jaeggi SM (2010). The relationship between n-back performance and matrix reasoning: Implications for training and transfer. Intelligence.

[32] Olesen PJ, Westerberg H, Klingberg T (2004). Increased prefrontal and parietal activity after training of working memory. Nat. Neurosci..

[33] Melby-Lervåg M, Redick TS, Hulme C (2016). Working memory training does not improve performance on measures of intelligence or other measures of “far transfer”: Evidence from a meta-analytic review. Perspect. Psychol. Sci..

[34] Redick TS (2013). No evidence of intelligence improvement after working memory training: A randomized, placebo-controlled study. J. Exp. Psychol. Gen..

[35] Onraedt T, Koster EHW (2014). Training working memory to reduce rumination. PLoS ONE.

[36] Li B (2016). Combined cognitive training vs. memory strategy training in healthy older adults. Front. Psychol..

[37] Gross AL (2012). Memory training interventions for older adults: A meta-analysis. Aging Ment. Health.

[38] Gelsomini F, Kanev K, Barneva RP, Walters L (2020). Technological enhancements of the method of loci for facilitating logographic language learning. J. Educ. Technol. Syst..

[39] Brehmer Y (2008). Comparing memory skill maintenance across the life span: Preservation in adults, increase in children. Psychol. Aging.

[40] Raz A (2009). A slice of π: An exploratory neuroimaging study of digit encoding and retrieval in a superior memorist. Neurocase.

[41] Maguire EA, Valentine ER, Wilding JM, Kapur N (2003). Routes to remembering: The brains behind superior memory. Nat. Neurosci..

[42] Werner-Seidler A, Dalgleish T (2016). The method of loci improves longer-term retention of self-affirming memories and facilitates access to mood-repairing memories in recurrent depression. Clin. Psychol. Sci..

[43] Bouffard N, Stokes J, Kramer HJ, Ekstrom AD (2018). Temporal encoding strategies result in boosts to final free recall performance comparable to spatial ones. Mem. Cogn..

[44] Kroneisen M, Makerud SE (2017). The effects of item material on encoding strategies: Survival processing compared to the method of loci. Q. J. Exp. Psychol..

[45] Bass WS, Oswald KM (2014). Proactive control of proactive interference using the method of loci. Adv. Cogn. Psychol..

[46] Brehmer Y, Li SC, Müller V, Von Oertzen T, Lindenberger U (2007). Memory plasticity across the life span: Uncovering children’s latent potential. Dev. Psychol..

[47] Rebok GW, Carlson MC, Langbaum JBS (2007). Training and maintaining memory abilities in healthy older adults: Traditional and novel approaches. J. Gerontol. B..

[48] Brehmer Y, Kalpouzos G, Wenger E, Lövdén M (2014). Plasticity of brain and cognition in older adults. Psychol. Res..

[49] Waris O, Soveri A, Laine M (2015). Transfer after working memory updating training. PLoS ONE.

[50] Szmalec A, Verbruggen F, Vandierendonck A, Kemps E (2011). Control of interference during working memory updating. J. Exp. Psychol. Hum. Percept. Perform..

[51] Strobach T, Salminen T, Karbach J, Schubert T (2014). Practice-related optimization and transfer of executive functions: A general review and a specific realization of their mechanisms in dual tasks. Psychol. Res..

[52] O’Hara R (2007). Long-term effects of mnemonic training in community-dwelling older adults. J. Psychiatr. Res..

[53] Swanson HL, Fung W (2016). Working memory components and problem-solving accuracy: Are there multiple pathways. J. Educ. Psychol..

[54] Schweizer S, Hampshire A, Dalgleish T (2011). Extending brain-training to the affective domain: Increasing cognitive and affective executive control through emotional working memory training. PLoS ONE.

[55] Lee Y, Lu M, Ko H (2007). Effects of skill training on working memory capacity. Learn. Instr..

[56] Yang P, Fan C, Wang M, Fogelson N, Li L (2017). The effects of changes in object location on object identity detection: A simultaneous EEG-fMRI study. Neuroimage.

[57] Hoffman P, Jefferies E, Lambon Ralph MA (2010). Ventrolateral prefrontal cortex plays an executive regulation role in comprehension of abstract words: Convergent neuropsychological and repetitive TMS evidence. J. Neurosci..

[58] Fliessbach K, Weis S, Klaver P, Elger CE, Weber B (2006). The effect of word concreteness on recognition memory. Neuroimage.

[59] Duñabeitia JA, Avilés A, Afonso O, Scheepers C, Carreiras M (2009). Qualitative differences in the representation of abstract versus concrete words: Evidence from the visual-world paradigm. Cognition.

[60] Pauligk S, Kotz SA, Kanske P (2019). Differential impact of emotion on semantic processing of abstract and concrete words: ERP and fMRI evidence. Sci. Rep..

[61] Montefinese M (2019). Semantic representation of abstract and concrete words: A minireview of neural evidence. J. Neurophysiol..

[62] Peng Y, Liu Y, Guo C (2019). Examining the neural mechanism behind testing effect with concrete and abstract words. NeuroReport.

[63] Perry C (2019). Working memory load affects early affective responses to concrete and abstract words differently: Evidence from ERPs. Cogn. Affect. Behav. Neurosci..

[64] Dahlin E, Neely AS, Larsson A, Backman L, Nyberg L (2008). Transfer of learning after updating training mediated by the striatum. Science.

[65] Morrison AB, Chein JM (2011). Does working memory training work? The promise and challenges of enhancing cognition by training working memory. Psychon. Bull. Rev..

[66] Vartanian O (2013). Working memory training is associated with lower prefrontal cortex activation in a divergent thinking task. Neuroscience.

[67] Chai WJ, Abd Hamid AI, Abdullah JM (2018). Working memory from the psychological and neurosciences perspectives: A review. Front. Psychol..

[68] Gathercole SE, Dunning DL, Holmes J, Norris D (2019). Working memory training involves learning new skills. J. Mem. Lang..

[69] Lövdén M, Brehmer Y, Li SC, Lindenberger U (2012). Training-induced compensation versus magnification of individual differences in memory performance. Front. Hum. Neurosci..

[70] Melby-Lervåg M, Hulme C (2013). Is working memory training effective? A meta-analytic review. Dev. Psychol..

[71] Lenhard, W. & Lenhard, A. *Calculation of Effect Sizes*. Retrieved from: https://www.psychometrica.de/effect_size.html. Dettelbach (Germany): Psychometrica. 10.13140/RG.2.2.17823.92329 (2016).

[72] Faul F, Erdfelder E, Buchner A, Lang AG (2009). Statistical power analyses using G*Power 3.1: Tests for correlation and regression analyses. Behav. Res. Methods.

[73] Gong Y (1983). Revision of Wechsler’s adult intelligence scale in China. Acta Psychol. Sin..

