# Supplementary material for: Dual n-back working memory training evinces superior transfer effects compared to the method of loci
Source: Sci Rep. 2021 Feb 4;11:3072. doi: 10.1038/s41598-021-82663-w (PMC7862396; doi:10.1038/s41598-021-82663-w)
Supplement: Supplementary file 1 — Supplementary Figure S1. [file 41598_2021_82663_MOESM1_ESM.docx]

**Title**

Dual n-back working memory training evinces superior transfer effects compared to the method of loci

Author names and affiliations

Wenjuan Li^1^, Qiuzhu Zhang^1^, Hongying Qiao^1^, Donggang Jin^1^, Ronald K Ngetich^1^, Junjun Zhang^1^, Zhenlan Jin^1*^, Ling Li^1*^

^1^ Key Laboratory for NeuroInformation of Ministry of Education, High-Field Magnetic Resonance Brain Imaging Key Laboratory of Sichuan Province, Center for Psychiatry and Psychology, School of Life Science and Technology, University of Electronic Science and Technology of China, Chengdu, 610054, China.

**Corresponding author**

E-mail: jinzl@uestc.edu.cn

liling@uestc.edu.cn


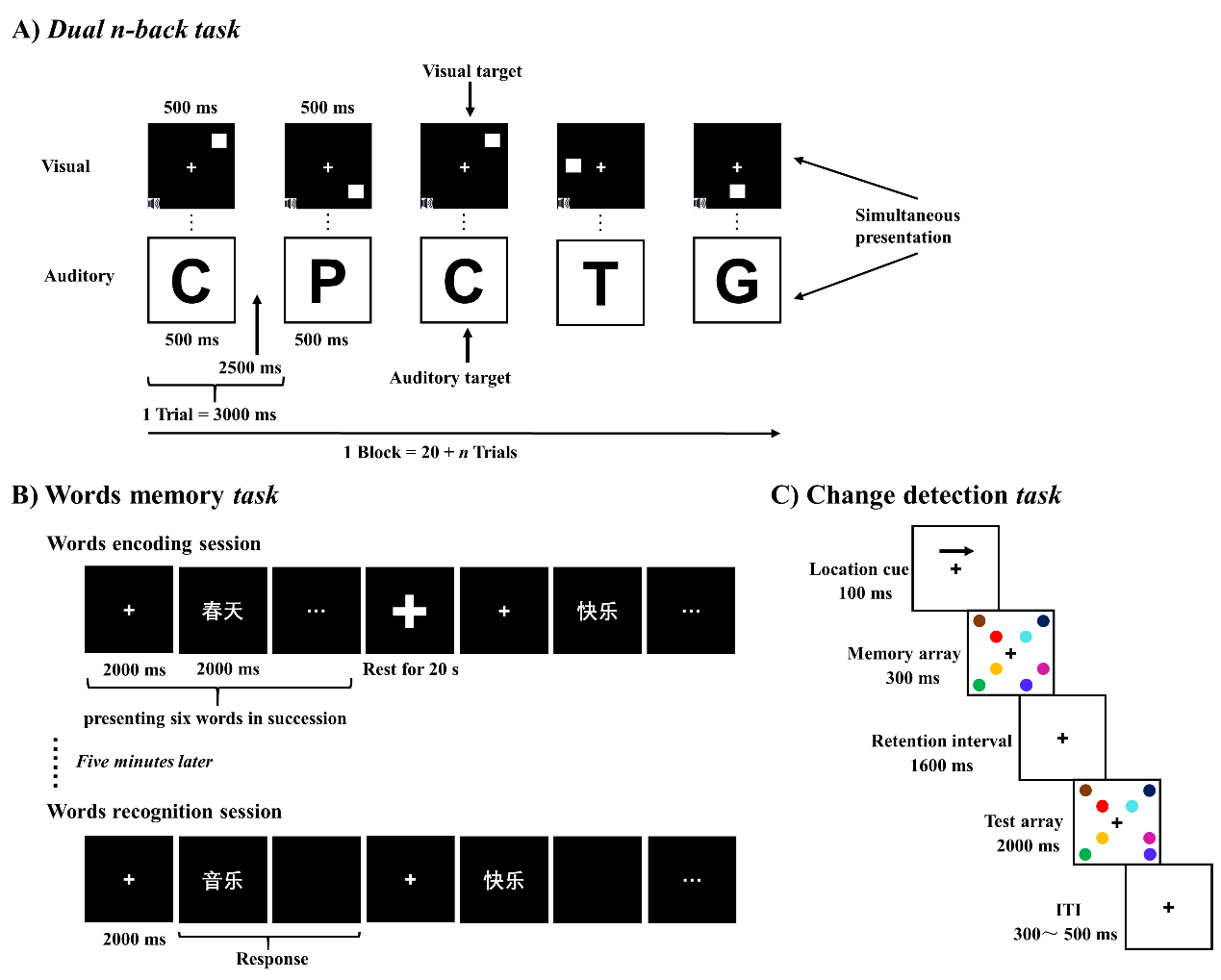


**Fig. S1.** Trial demonstration of task paradigms. A) The dual n-back task. The diagram depicts the condition of 2-back task. B) The words memory task. All words are written in Mandarin in the task. C) The change detection task. The figure illustrates a 4 load condition.
